# The Correlates of Cognitive Status Among Older Americans Living in the Community: Does Gender Make a Difference?

**DOI:** 10.1093/geroni/igaa057.206

**Published:** 2020-12-16

**Authors:** Ethan Siu Leung Cheung, Ada Mui, Seth Hoffman

**Affiliations:** 1 Columbia University, Bronx, New York, United States; 2 Columbia University, New York, New York, United States; 3 Columbia University, Rego Park, New York, United States

## Abstract

Utilizing the data in National Social Life, Health and Aging Project (n = 3,104; 54% female), the study examined the predictors of cognitive impairments in terms of community harmony, community safety, frequency of neighbor contacts, depression, and demographic factors. Bivariate analyses suggest that there were no gender differences in cognitive status (Mean of MoCA Short Form = 9.89; SD = 3.33); nor were there gender differences in age (mean age = 72.95; SD=8.29), ethnic composition (76.1% whites; 15.3% Blacks, 8.6% Asian), community harmony, community safety, frequency of neighbor contacts. On the other hand, men had more education and income than women. Psychologically, older women reported higher level of stress and depression scores than older men. Multiple regression results show that gender has a significant independent effect and joint effects with stressors and community factors in explaining cognitive impairments. Parallel regression analyses for each gender group were conducted and models were significant (P < .0001). There were common predictors of cognitive impairments for the two groups but variables had differential impacts on older men and older women. Specifically, IADL had stronger effect on older men than on older women in predicting cognitive impairments (b = -.23 vs. b=-.10); perceived community harmony had stronger impact on older women in explaining their cognitive status (b = .26 vs. b=.22); older women’s cognitive status benefitted more from perceived community safety than older men (b = .61 vs. b=.43). Regardless of gender, older Whites scored higher than Black and Asian elders in their cognition scores.

